# Commentary: The use and misuse of life course models

**DOI:** 10.1093/ije/dyw101

**Published:** 2016-10-06

**Authors:** Rebecca Hardy, Kate Tilling

**Affiliations:** 1 ^1^MRC Unit for Lifelong Health and Ageing at UCL, University College London, London, UK; 2 ^2^MRC Integrative Epidemiology Unit at the University of Bristol and School of Social and Community Medicine, University of Bristol, Bristol, UK

There is increasing emphasis in medical research on fetal and childhood antecedents of disease, and how these interact with other exposures throughout the life course to influence later-life conditions. As outlined by Ben-Shlomo *et al.*,^1^ answering questions about the relative importance of aspects and timing of growth, behaviour and health status for longer-term outcomes requires appropriate analyses of longitudinal data.

Analysis of such data inevitably poses statistical challenges due to the complex temporal relationships between multiple factors across life.^2^^,^^3^ Analyses must account for dependencies between repeated observations on the same person: methods to do this (e.g. random effects models^4^) are now widely available in standard statistical software packages. However, when repeated measures are taken frequently, there is likely to be serial autocorrelation among the measurements (greater correlation among measurements closer together in time), which requires more complex models. Where there are repeated measures of exposures related to a later-life outcome, standard regression models may be affected by multicollinearity among the repeated exposures. Measurement error may vary over time (e.g. absolute measurement error in weight will be larger in adulthood than childhood), which will need to be taken into account in any analysis. There will also usually be dropout over time due to death, illness or refusal to participate, which will limit the sample size and may result in bias in complete case analyses.

When the initial life course models were proposed, statistical methods for addressing questions about repeated exposures and outcomes were under-developed. Life course epidemiology stimulated research on the methodology to be able to better address such research questions. The focus of life course methodology, initially at least, was in the analysis of repeated measures of the same exposure—and in many cases, specifically for the analysis of growth and its association with later outcomes. Simpler approaches are appropriate for a small number of repeated measures of an exposure, each recorded at the same age for all individuals (e.g. weight measured at 2, 4 and 6 years of age). Early on in life course epidemiology, one method was to plot average z-scores over time for the two groups formed by a dichotomous outcome. These z-score plots can be misinterpreted as growth trajectories,^5^ whereas in fact they show a series of cross-sectional associations of exposure with outcome.

Further developments led to the use of regression models for the outcome, including various parameterizations of the exposure, depending on whether conditioning on previous measures of the same exposure, or using observed measures or change scores.^2^ In turn, this led to awareness of the dangers of conditioning on variables which are on the causal pathway from exposure to outcome—such as including adult weight in models relating birthweight to adult disease.^6^ More recently, increasingly complex approaches such as multilevel models or latent variable models have been used to describe patterns of change and relate these patterns to various health outcomes.^5^ Essentially, all approaches aim to relate changes in an exposure such as body size to a later outcome, with some of the methods, such as multilevel models, parameterizing average and individual change, and others, in particular latent class models, describing subpopulations with different patterns of growth. These types of models have been extended to relate trajectories of several repeatedly measured variables to each other, for example to relate repeated measures of mean arterial pressure to simultaneous weight gain during pregnancy.^7^

A simple structured modelling approach using regression was proposed in order to distinguish a critical period model from an accumulation model when relating a repeated binary exposure to an outcome.^8^ It is being increasingly applied in the epidemiological literature, mainly in relation to lifetime socioeconomic position as in the original example,^9–12^ but also to other repeated binary exposures such as overweight/obesity.^13^^,^^14^ The original example involved occupational social class (categorized into manual and non-manual) at three ages selected to represent different periods of the life course—childhood, early adulthood and midlife. Each alternative life course model is tested against the saturated model (where all eight possible trajectories have a different mean outcome) using an F-test. Larger *P*-values indicate that the more parsimonious model fits the data as well as the saturated model and thus can be deemed a good fit. Accumulation and critical period models were considered alongside social mobility. Social mobility is not a life course model as such, but is a concept of particular interest to social scientists, and the operationalization of social mobility remains contentious. Additional variations on these original models have subsequently been considered.^10^^,^^14^

The advantage of the approach is that it provides a way to compare a set of pre-specified models and forces a clear specification of the models of interest. Analyses that only consider the association of a cumulative score with an outcome may be misleading and conclude that there is evidence for accumulation, as a cumulative score can capture the effect of a critical period.^8^ Although, as Ben-Shlomo *et al.* now note, a sensitive period and a critical period are more sensibly viewed as subsets of an accumulation model rather than being distinct, when considering the same exposure over the life course. The selection of the best fitting model based on *P*-values is not ideal and has proved challenging in practice as, for example, there are situations where more than one model fits as well as the saturated model.

An alternative approach where model selection is based on a least absolute shrinkage and selection operator (lasso), which does not rely on significance testing, has recently been developed.^15^ Structural equation models have also been used to test the fit to the data of competing hypotheses.^16^ The ability to distinguish between the various models is limited by the variability in trajectories within any dataset, and the timing and spacing of the measurement points. In the original paper, measures were selected to cover three relatively equally spaced but distinct periods of the life course. In an application with only childhood and adult socioeconomic position (SEP), the adult SEP exposure covered a considerably longer period of time than the childhood measure.^9^

Another example used three equally spaced measures of SEP, but covering only a relatively short period of the life course from early to late midlife (ages 40, 50 and 60) where fewer changes in SEP might be expected.^12^ It is less clear whether a critical period would be a potential model in this example, compared with when considering more distinct periods of the life course. The approach has been applied using differing markers of SEP at different ages. Education, for example, was used as an early adult marker and occupational social class for childhood and midlife.^10^ This first raises the practical challenge of dichotomizing different measures of SEP in a comparable way. Second, there is the possibility that it may be the particular measure, as opposed to the time period that it is representing, that is more (or less) informative, and mediation analysis may be more appropriate in such cases. Whether using the same or different indicators of SEP, the structured life course modelling approach has generally been applied without acknowledgement of the fact that adult SEP is a potential mediator of the relationship between early life SEP and the outcome, and that thus conditioning on adult SEP may induce collider bias. Modern methods of mediation analysis have been developed,^17^ and are informed by causal inference thinking, as outlined in a commentary in this issue.^18^

Often in life course epidemiology the question of interest is not limited to repeated measures of the same exposure. Indeed, the original accumulation models were defined in terms of different exposures even though they have generally been applied in the context of the same exposure.^19^ A recent paper examined associations between birthweight, development in infancy, socioeconomic position and depression in adolescence and adulthood. Structural equation models were used to estimate the size of the relationships among all these variables, and examine direct and indirect effects on repeated measures of the outcome (depression).^20^ A drawback to structural equation models is that they can be relatively complex to fit, and are easier to use with data measured on regular occasions (e.g. six waves of a cohort study) than with data measured irregularly (e.g. routine measures of weight taken during infancy). Structural equation models are not inherently ‘causal’; a given model may not be able to distinguish the hypothesis that A causes B from the hypothesis that B causes A, for example. However, parameters from structural equations can often be interpreted in a causal framework, and have been used to examine mediation.^21^ Structural equation models are able to take into account known measurement error, latent variables, repeated measures of exposures, covariates and outcomes, and complex associations. For example, exposure at one time point could influence a confounder at the next, which could in turn influence later exposure.^22^ This type of confounding, often known as time-varying confounding, can also be examined using other methods such as marginal structural models.^17,18^

Although complex methods are increasingly used in epidemiology, they are often too limited to accommodate the complexities of life course analyses. Their typical use is uncritical: results are often reported without making model assumptions and study hypotheses explicit. Choice of method often depends more on the study design than the question being asked, and dependence of conclusions on assumptions such as missing data mechanisms, measurement error and absence of confounding is seldom examined. In particular, all the models described here depend crucially on the assumption of no unmeasured confounding, and sensitivity to this, or to choice of modelling framework, is infrequently investigated. In order to support the investigation of increasingly complex life course hypotheses, we need corresponding development in statistical methods and their use. Choice of methods should be guided by the question of interest, thus requiring understanding of the underlying biology and proposed causal mechanisms. Burgeoning areas of focus include high-dimensional data (epigenetics metabolomics), and use of intensively collected data to measure phenotypes more accurately (e.g. use of accelerometers, GPS data). We need to further develop ways to integrate all these types of data into one analytical framework.

**Conflict of interest:** None

## References

[1] Ben-Shlomo Y Cooper R Kuh D. The last two decades of life course epidemiology, and its relevance for research on ageing. Int J Epidemiol 2016;45:973–88. 27880685 10.1093/ije/dyw096 PMC5841628

[2] De Stavola B Nitsch D dos Santos Silva I Statistical issues in life course epidemiology. Am J Epidemiol 2006;163:84–96. 16306313 10.1093/aje/kwj003

[3] Wills A Tilling K. Modelling repeat exposures: some examples from life course epidemiology In: Kuh D Cooper R Hardy R Richards M Ben-Shlomo Y (eds). A Life Course Approach to Healthy Ageing. Oxford, UK: Oxford University Press, 2014.

[4] Goldstein H Browne W Rasbash J. Multilevel modelling of medical data. Stat Med 2002;21:3291–315. 12375305 10.1002/sim.1264

[5] Tu Y-K Tilling K Sterne JA Gilthorpe MS. A critical evaluation of statistical approaches to examining the role of growth in the developmental origins of health and disease. Int J Epidemiol 2013;42:1327–39. 24038715 10.1093/ije/dyt157

[6] Tu Y-K West R Ellison GTH Gilthorpe MS. Why evidence for the fetal origins of adult disease might be a statistical artifact: the ‘reversal paradox’ for the relation between birth weight and blood pressure in later life. Am J Epidemiol 2005;161:27–32. 15615910 10.1093/aje/kwi002

[7] Macdonald-Wallis C Lawlor D Palmer T Tilling K. Multivariate multilevel spline models for parallel growth processes: application to weight and mean arterial pressure in pregnancy. Stat Med 2012;21:3147–64. 10.1002/sim.5385 PMC3569877 22733701

[8] Mishra GD Nitsch D Black S De Stavola B Kuh D Hardy R. A structured approach to modelling the effects of binary exposure variables across the life course. Int J Epidemiol 2009;38:528–37. 19028777 10.1093/ije/dyn229 PMC2663717

[9] Birnie K Martin RM Gallacher J, Socio-economic disadvantage from childhood to adulthood and locomotor function in old age: a lifecourse analysis of the Boyd Orr and Caerphilly prospective studies. J Epidemiol Community Health 2011;65:1014–23. 20644236 10.1136/jech.2009.103648 PMC3381706

[10] Robertson T Popham F Benzeval M. Socioeconomic position across the lifecourse and allostatic load: data from the West of Scotland Twenty-07 cohort study. BMC Public Health 2014;14:184. 24555560 10.1186/1471-2458-14-184 PMC3942053

[11] Elwell-Sutton TM Jiang CO Zhang WS Socioeconomic influences at different life stages on health in Guangzhou, China. Soc Sci Med 2011;72:1884–92. 21550152 10.1016/j.socscimed.2011.03.041

[12] Padyab M Norberg M. Socioeconomic inequalities and body mass index in Västerbotten County, Sweden: a longitudinal study of life course influences over two decades. Int J Equity Health 2014;13:35. 24884742 10.1186/1475-9276-13-35 PMC4018623

[13] Wills AK Black S Cooper R Life course body mass index and risk of knee osteoarthritis at the age of 53 years: evidence from the 1946 British birth cohort study. Ann Rheum Dis 2012;71:655–60. 21979003 10.1136/ard.2011.154021 PMC3329229

[14] Murray ET Hardy R Hughes A Overweight across the life course and adipokines, inflammatory and endothelial markers at age 60-64 years: Evidence from the 1946 birth cohort. Int J Obes 2015;39:1010-18. 10.1038/ijo.2015.19 PMC4433551 25676237

[15] Smith AD Heron J Mishra G Gilthorpe MS Ben-Shlomo Y Tilling K Model selection of the effect of binary exposures over the life course. Epidemiology 2015;26:719–26. 26172863 10.1097/EDE.0000000000000348 PMC4521897

[16] Juneau CE Sullivan A Dodgeon B Cote S Ploubidis GB Potvin L. Social class across the life course and physical activity at age 34 years in the 1970 British birth cohort. Ann Epidemiol 2014;24:641–47. 25127740 10.1016/j.annepidem.2014.06.096

[17] Richiardi L Bellocco R Zugna D. Mediation analysis in epidemiology: methods, interpretation and bias. Int J Epidemiol 2013;42:1511-19. 24019424 10.1093/ije/dyt127

[18] De Stavola BL Daniel RM Incorporating concepts and methods from causal inference into life course epidemiology. Int J Epidemiol 2016;45:1006–10. 27880691 10.1093/ije/dyw103 PMC5965915

[19] Kuh D Ben-Shlomo Y Lynch J Hallqvist J Power C. Life course epidemiology. J Epidemiol Community Health 2003;57:778–83. 14573579 10.1136/jech.57.10.778 PMC1732305

[20] Colman I Jones PB Kuh D Weeks M Naicker K Croudace TJ. Early development, stress and depression across the life course: pathways to depression in a national British birth cohort. Psychol Mede 2014;44:2845–54. 10.1017/S0033291714000385 25066933

[21] De Stavola BL Daniel RM Ploubidis GB Micali N. Mediation analysis with intermediate confounding: structural eqation modeling viewed through the causal inference lens. Am J Epidemiol 2015;181:64–80. 25504026 10.1093/aje/kwu239 PMC4383385

[22] Kaakinen M Sovio U Hartikainen ALPA Life course structural equation model of prenatal and postnatal growth on adult blood pressure. J Epidemiol Community Health 2014;68:1161–67. 25108017 10.1136/jech-2013-203661

